# Enhancing Structural and Interfacial Stability of NaNi_1/3_Mn_1/3_Fe_1/3_O_2_ Cathodes via Sb^3+^ Doping for Sodium Ion Batteries

**DOI:** 10.3390/nano15201575

**Published:** 2025-10-16

**Authors:** Yong Liu, You Shi, Mengjie Zhang, Dan Sun, Huanhuan Li, Haiyan Wang, Yougen Tang

**Affiliations:** 1 Hunan Provincial Key Laboratory of Chemical Power Sources, College of Chemistry and Chemical Engineering, Central South University, Changsha 410083, China; 202302050@csu.edu.cn (Y.L.); 232301033@csu.edu.cn (Y.S.); 242306059@csu.edu.cn (M.Z.); sundan4330@csu.edu.cn (D.S.); 2 Sunwoda Mobility Energy Technology Co., Ltd., Shenzhen 518100, China; 3 School of Chemistry and Chemical Engineering, Henan Normal University, Xinxiang 453007, China; 2017076@htu.edu.cn

**Keywords:** cathode material, NaNi_1/3_Mn_1/3_Fe_1/3_O_2_, grain morphology regulation, homogenizing stress distribution

## Abstract

O3-type NaNi_1/3_Mn_1/3_Fe_1/3_O_2_ (NFM) cathodes for sodium-ion batteries face critical challenges of sluggish Na^+^ diffusion and structural degradation during cycling. In this study, we implement an Sb^3+^ doping strategy that enhances structural stability and interfacial stability by modulating the NFM grain morphology to promote densification of primary particles and shorten Na^+^ migration paths. The optimized Sb-doped NFM1Sb (1%mol Sb) cathode exhibits excellent electrochemical performance, achieving 86.48% capacity retention after 200 cycles at 1 C and a high rate capability of 122.2 mAh g^−1^ at 5 C. These improvements are attributed to the alleviation of stress concentration and suppression of microcrack formation during cycling. This work demonstrates the critical role of grain morphology regulation through heavy-metal doping in developing long-life and high-rate SIBs, providing a viable pathway toward next-generation energy storage systems.

## 1. Introduction

The commercialization of sodium-ion batteries (SIBs) has gained considerable momentum since 2023, marked by their successful integration into light electric vehicles and grid-scale energy storage systems [1,2,3,4,5,6,7,8]. To further expand their market penetration, it is imperative to enhance key performance metrics, including cost-effectiveness, operational safety, and energy density [9,10,11,12]. Among the available cathode materials are layered transition metal oxides, Prussian blue analogs, and polyanionic compounds. O3-type Ni-Fe-Mn-based oxides (e.g., NaNi_1/3_Mn_1/3_Fe_1/3_O_2_, NFM) have emerged as particularly promising candidates [13,14,15]. These materials benefit from their compatibility with existing lithium-ion battery manufacturing infrastructure and deliver a high specific capacity of approximately 140 mAh g^−1^ [16,17,18,19].

However, the widespread adoption of NFM cathodes is challenged by two inherent limitations: (i) sluggish Na^+^ diffusion kinetics, which lead to unsatisfactory rate performance, and (ii) structural degradation caused by transition metal dissolution and interfacial side reactions, resulting in rapid capacity fading [17,20,21,22,23,24,25]. Recent advances in doping strategies have shown potential in addressing these issues. For instance, La^3+^ bulk doping has been reported to facilitate Na^+^ mobility and mitigate lattice strain [26]. Inspired by the successful application of heavy-metal dopants (such as Mo, Sb, and Nb) in stabilizing lithium-ion cathode materials [27,28,29,30], we propose a strategy of Sb^3+^ lattice engineering to precisely regulate the grain morphology of NFM. This approach simultaneously achieves two critical objectives: (i) densification of primary particles via crystallographic orientation control, and (ii) reduction of Na^+^ migration barriers through particle size refinement.

In this study, we demonstrate that Sb^3+^ doping significantly enhances the electrochemical performance of O3-type NFM cathodes. The optimized Sb-doped NFM (NFM1Sb) exhibits a remarkable capacity retention of 86.48% after 200 cycles at 1 C, along with a high reversible capacity of 122.2 mAh g ^−1^ even at 5 C. The tailored grain morphology effectively alleviates stress concentration during repeated sodium insertion and extraction, thereby substantially inhibiting the formation of microcracks. The synergy between improved structural stability and enhanced Na^+^ diffusion kinetics establishes a robust foundation for developing long-life and high-performance SIBs. This study underscores the crucial importance of grain morphology regulation in the design of advanced SIB cathodes. The combination of Sb^3+^ doping with precise microstructure control offers a novel and effective strategy for achieving durable and high-rate sodium-ion batteries, thereby facilitating their deployment in next-generation energy storage applications.

## 2. Materials and Methods

### 2.1. Material Preparation

Sb-doped NaNi_1/3_Fe_1/3_Mn_1/3_O_2_ cathode materials were synthesized through a combined approach of ball milling and high-temperature solid-state reaction. Initially, the commercial hydroxide precursor Ni_1/3_Fe_1/3_Mn_1/3_(OH)_2_ (Hunan Zhongwei New Material) was calcined in a muffle furnace under ambient air atmosphere. The temperature was raised to 550 °C at a heating rate of 3 °C min^−1^, maintained for 5 h, and then naturally cooled to obtain the oxide precursor Ni_1/3_Fe_1/3_Mn_1/3_O_x_. Subsequently, the obtained oxide precursor was mixed with Na_2_CO_3_ and nano-Sb_2_O_3_ according to molar ratios of Na:M:Sb = 1:1.02:x (x = 0, 0.005, 0.01, 0.02). The mixture was subjected to mechanical ball milling in a zirconia jar with a ball-to-powder mass ratio of 1:1 at 300 rpm for 3 h. The homogenized powder was then sintered in a dry air-filled muffle furnace. The temperature was elevated to 900 °C at 3 °C min^−1^ and held for 15 h, followed by natural cooling to room temperature to obtain the final cathode materials. The resulting products were designated as NFM (x = 0), NFM0.5Sb (x = 0.005), NFM1Sb (x = 0.01), and NFM2Sb (x = 0.02) based on the Sb doping concentration.

### 2.2. Material Characterization and Electrochemical Analysis

All detailed information for the material characterization, computational methods, and electrochemical measurements is shown in the Supplementary Information.

## 3. Results and Discussion

**Figure 1:**
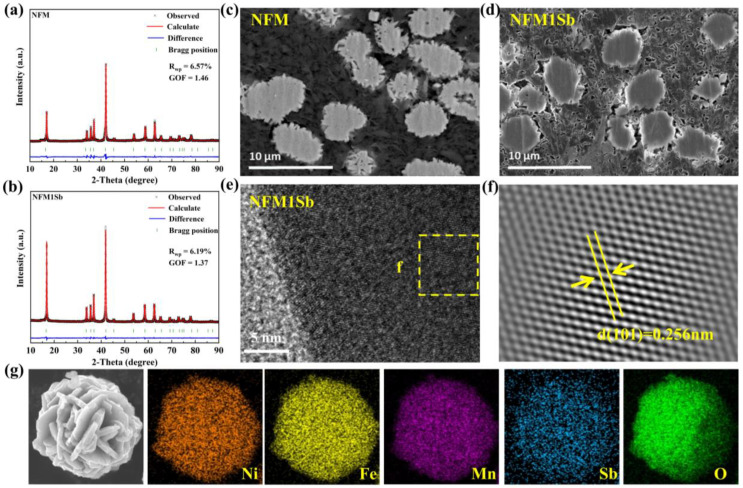
The refined XRD patterns of (**a**) NFM and (**b**) NFM1Sb. Cross-sectional SEM images of (**c**) NFM and (**d**) NFM1Sb. (**e**) HRTEM image of NFM1Sb. (**f**) FFT mode. (**g**) The SEM-EDS mapping images of NFM1Sb.

NFM and Sb-doped NFM samples were synthesized via mechanical mixing followed by a high-temperature solid-state sintering process. Figure S1 displays the morphology images of pristine sample and samples with different doping contents. As seen, all samples maintain spherical morphology with ∼5 μm secondary particles comprising aggregated primary grains. Sb doping induces progressive surface densification of secondary particles across doping levels, demonstrating effective morphological control through lattice modification. XRD patterns of the four samples are shown in Figure S2; all samples exhibit phase-pure O3-type structure (R-3m space group). Compared with NFM, the (003) and (104) peaks of the Sb-doped samples shift towards a lower degree, suggesting a change in crystal structure. Rietveld refinement was conducted on all four materials to obtain precise unit cell parameters (Figure 1a,b and Figure S3), with the refined results summarized in Table S1. The lattice parameters exhibit systematic expansion with increasing Sb doping content, which can be attributed to the substitution of smaller transition metal ions (Ni^3+^: 0.56 Å, Fe^3+^: 0.645 Å, Mn^4+^: 0.53 Å) by the larger Sb^3+^ (0.76 Å) [31,32]. Specifically, the a-axis expansion corresponds to increased interlayer spacing within the transition metal oxide (TMO_2_) layers, while the c-axis elongation arises from enhanced electrostatic repulsion between oxygen atoms in adjacent TMO_2_ layers. This structural modification significantly enlarges the sodium layer (NaO_2_) spacing, thereby expanding sodium-ion diffusion channels and potentially improving ionic transport kinetics [33]. The coordinated lattice expansion demonstrates effective structural modulation through Sb doping, which likely contributes to enhanced electrochemical performance by facilitating Na^+^ mobility within the layered framework.

To further investigate the influence of Sb doping on the internal morphology of sample particles, NFM and NFM1Sb samples were subjected to argon ion beam polishing followed by cross-sectional SEM characterization. As shown in Figure 1c,d, NFM exhibits internal structures composed of loosely aggregated nanosized primary particle agglomerates, whereas NFM1Sb demonstrates significantly reduced porosity and enhanced structural compactness in its cross-sectional profile. These structural observations confirm that Sb doping effectively modifies the internal microstructure of cathode particles, which would be beneficial to reduce electrolyte penetration and side reactions. Figure S4 presents the cross-sectional line-scan EDS mapping of NFM1Sb, verifying that Sb^3+^ ions are homogeneously distributed throughout the particle interior. Representative high-resolution TEM images of NFM1Sb (Figure 1e) and NFM (Figure S5) reveal well-defined lattice fringes, with the corresponding fast Fourier transform (FFT) pattern (Figure 1f) demonstrating characteristic diffraction spots. The measured lattice fringe spacing of 0.256 nm corresponds to the (101) crystallographic plane, confirming the layered structure of the material and aligning with the previous XRD analysis.

**Figure 2:**
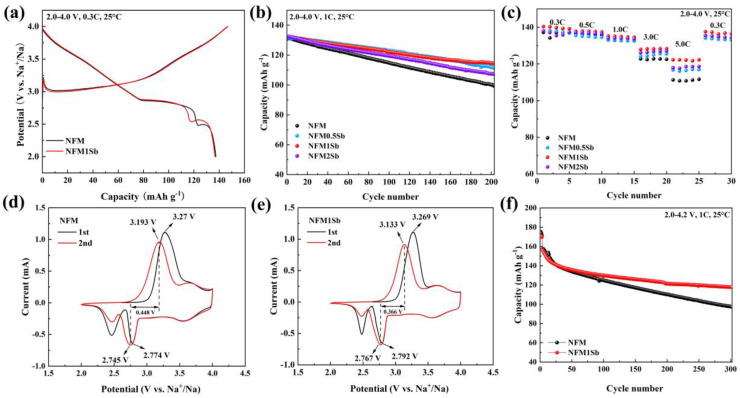
(**a**) Initial charge–discharge curves at 0.3 C. (**b**) Cycling stability at 1 C. (**c**) Rate performance from 0.3 C to 5 C. CV curves at 0.02 mV s^−1^ of (**d**) NFM and (**e**) NFM1Sb. (**f**) Cycling stability at 1C in the voltage range of 2.0–4.2 V at 25 °C.

To evaluate the impact of Sb doping on the electrochemical performance of NaNi_1/3_Mn_1/3_Fe_1/3_O_2_ material, all samples were subjected to constant current electrochemical performance testing in a half-cell system using metallic sodium as the anode. Figure 2a shows the charge–discharge curves of NFM and NFM1Sb cathodes under the voltage range of 2.0 V to 4.0 V at 0.3 C (1 C = 150 mA g^−1^). The platform around 3.0 V corresponds to the two-phase coexistence transition area of the O3-P3 phase transformation, indicating a solid-solution reaction occurring during the charging process [34]. The sloping section above 3.2 V to 4.0 V corresponds to the P3 phase. Figure 2b compares the long cycle stability of the four samples at a current density of 1 C, and the NFM1Sb displays outstanding cycling stability over 200 cycles with 86.48% capacity retention and higher than NFM under the same testing conditions. Additionally, the cycling stability of NFM0.5Sb and NFM2Sb is also higher than that of NFM, with 200-cycle capacity retention rates of 84.86% (NFM0.5Sb), 81.04% (NFM2Sb), and 75.99% (NFM), respectively. At the same time, the charge–discharge curves of NFM and NFM1Sb at different cycle numbers at 1 C were compared. As observed from Figure S6, the voltage platform of NFM1Sb decreases more slowly compared to NFM, indicating that Sb doping helps to suppress the voltage hysteresis during the cycling process. The NFM1Sb cathode manifests much better rate performance than the NFM under the same current densities from 0.3 C to 5 C (Figure 2c), demonstrating good reversibility [35]. Moreover, NFM0.5Sb and NFM2Sb also show higher capacities at higher rates than NFM. This indicates that Sb doping can significantly improve the rate performance of NFM.

Cyclic voltammetry (CV) tests were conducted on NFM and NFM1Sb within a voltage range of 2.0–4.0 V. As shown in Figure 2d,e, NFM1Sb cathode exhibits a reduced potential difference (ΔV) between oxidation and reduction peaks compared to NFM, decreasing from 0.448 V to 0.366 V at a scan rate of 0.2 mV s^−1^, which aligns with its enhanced specific capacity and improved reversibility. Furthermore, the voltage range was extended to 2.0–4.2 V to further study the impact of Sb doping on the electrochemical performance of the material. As shown in Figure 2f, NFM1Sb still demonstrates better cycling stability, with a capacity retention of 75.5% after 300 cycles at a current density of 1 C, while the NFM only has a capacity retention of 62%. Therefore, Sb doping can improve cycling stability under high rates and high voltage. The commercial potential of NFM1Sb cathode was systematically evaluated through the full sodium-ion cells employing NFM1Sb as the cathode paired with commercial hard carbon anodes, as depicted in Figure S7a. The mass ratio between cathode and anode was precisely optimized according to their respective half-cell electrochemical performances (Figure S7b) coupled with charge-balance considerations. The full cell demonstrates outstanding cycling stability (Figure S7c), achieving an energy density of 349.23 Wh kg^−1^ (cathode-mass-based) with 121.5 mAh g^−1^ discharge capacity at 1 C rate, while retaining 81.8% capacity after 100 cycles. The comprehensive performance metrics establish NFM1Sb as a viable cathode material for sodium-ion batteries, demonstrating exceptional commercial viability.

**Figure 3:**
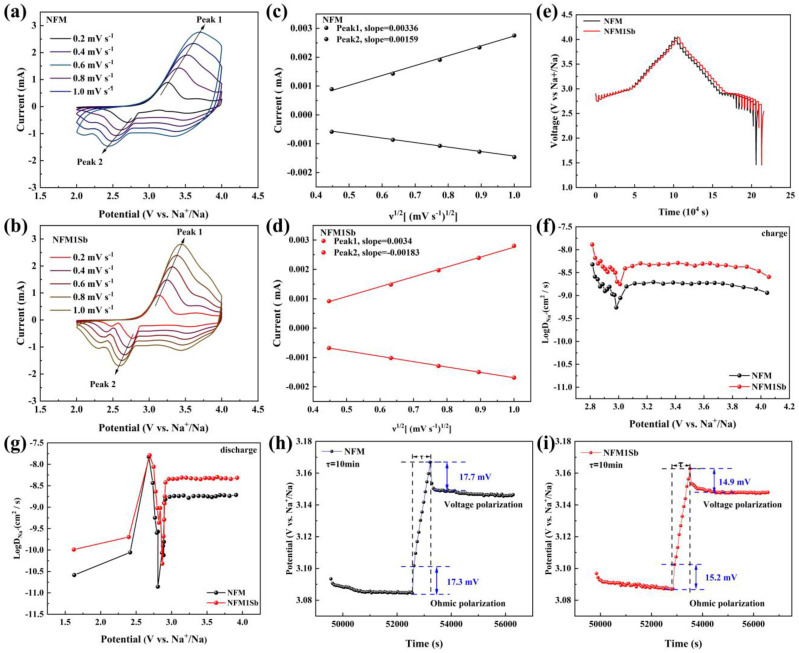
The CV curves of (**a**) NFM and (**b**) NFM1Sb at different scan rates ranging from 0.2 to 1 mV s^−1^. Linear fitting of (**c**) NFM and (**d**) NFM1Sb at the oxidation and reduction peaks for the CV test. (**e**) GITT charge–discharge curves of NFM and NFM1Sb. Calculated sodium ion diffusion coefficients for NFM and NFM1Sb during (**f**) charge and (**g**) discharge. Voltage polarization and ohmic polarization during charging for (**h**) NFM and (**i**) NFM1Sb.

CV profiles at varying scan rates (0.2–1.0 mV s^−1^, Figure 3a,b) reveal that NFM1Sb maintains more symmetric and well-defined redox peaks even at elevated scan rates, demonstrating superior rate capability attributed to Sb substitution. Quantitative analysis of the linear relationship between peak current and the square root of scan rate indicates that higher slopes correspond to larger apparent Na^+^ diffusion coefficients (D_Na_^+^). Notably, as illustrated in Figure 3c,d, NFM1Sb exhibits steeper slopes during both charge and discharge processes compared to NFM, confirming that Sb doping significantly enhances the apparent Na^+^ diffusion kinetics, thereby contributing to improved electrochemical performance. The sodium-ion diffusion kinetics were systematically investigated through galvanostatic intermittent titration technique (GITT) measurements to determine Na^+^ diffusion coefficients. The GITT curves for NFM and NFM1Sb samples are presented in Figure 3e. Quantitative analysis of diffusion coefficients (Figure 3f,g) reveals a substantial enhancement in Na^+^ mobility [36], with NFM1Sb displaying a diffusion coefficient of 2.98 × 10^−9^ cm^2^ s^−1^, nearly three times higher than that of NFM (1.08 × 10^−9^ cm^2^ s^−1^). This remarkable improvement in ionic transport kinetics can be attributed to Sb doping-induced structural modifications that effectively enlarge sodium-ion migration channels, as evidenced by the expanded interlayer spacing observed in previous XRD and Rietveld refinement analyses. Figure 3h,i demonstrate that NFM1Sb exhibits significantly reduced ohmic polarization and lower average voltage polarization compared to pristine NFM. To further verify the conductivity enhancement induced by Sb doping, electrochemical impedance spectroscopy (EIS) was performed on NFM and NFM1Sb electrodes after 1 cycle and 200 cycles (Figure S8). As seen, NFM1Sb exhibits much lower charge transfer resistance (R_ct_) compared to pristine NFM, demonstrating improved electrical conductivity that aligns with the enhanced electrochemical performance observed in CV and GITT analyses. This consistent reduction in interfacial resistance across cycling stages confirms the structural stability and sustained conductivity benefits conferred by Sb doping.

**Figure 4:**
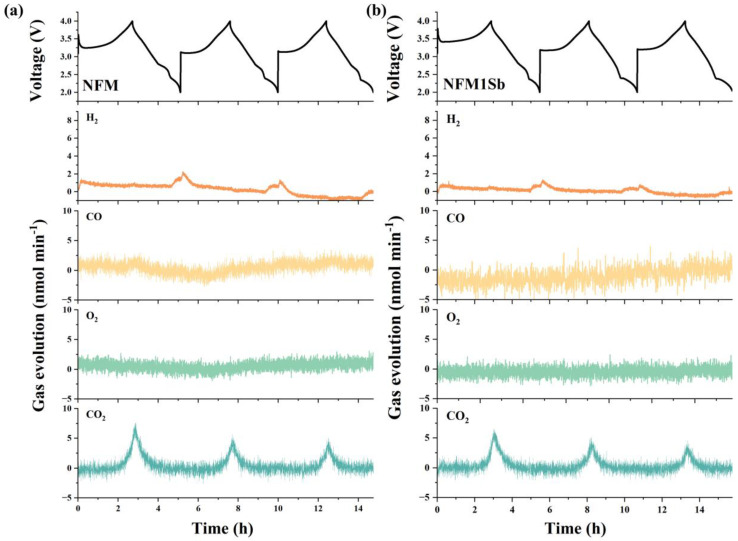
DEMS test results of (**a**) NFM and (**b**) NFM1Sb at 2.0–4.0 V.

**Figure 5:**
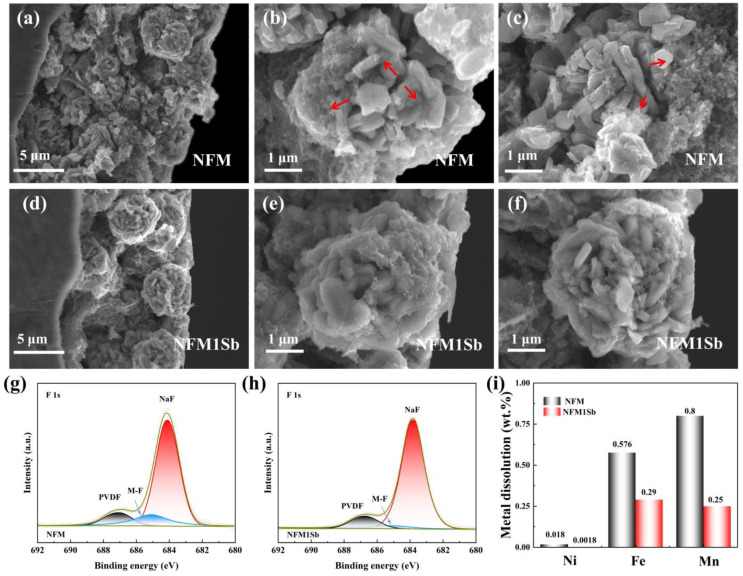
SEM images of (**a**–**c**) NFM and (**d**–**f**) NFM1Sb after 200 cycles. XPS spectra of F1s for (**g**) NFM and (**h**) NFM1Sb after 100 cycles. (**i**) Metal dissolution plots of Ni, Fe, and Mn for NFM and NFM1Sb after 100 cycles at 25 °C.

In situ differential electrochemical mass spectrometry (DEMS) analysis (2.0–4.0 V) revealed gas evolution during cycling. Initial CO_2_ peaks originate from residual surface carbonates (Figure 4), decreasing in subsequent cycles due to suppressed interfacial reaction [37]. Concurrent H_2_ release stems from high-voltage solvent oxidation (R-H^+^ formation) and anode reduction [38]. Sb-doped NFM1Sb exhibits reduced CO_2_/H_2_ emissions versus pristine NFM, attributed to diminished carbonate residues and limited electrolyte contact [39], demonstrating enhanced interfacial stability and intrinsic safety. Post-cycling morphological evolution of electrodes was analyzed through SEM characterization (Figure 5a–f). The cycled NFM cathode exhibits pronounced microcracks resulting from repetitive unit cell volume variations during Na^+^ intercalation/deintercalation processes. These structural defects create penetration pathways for electrolyte infiltration, accelerating parasitic reactions between the electrolyte and fresh active material within particle interiors. In contrast, the NFM1Sb electrode maintains crack-free surfaces with preserved particle integrity, demonstrating that the compact internal architecture imparted by Sb doping effectively mitigates stress-induced fracture propagation through enhanced structural stability during prolonged cycling. The superior structural integrity of NFM1Sb constitutes the key mechanism for its exceptional capacity retention, achieved by suppressing electrolyte penetration and blocking subsequent degradation pathways of active materials. As revealed by the XPS analysis of the cycled electrodes (Figure 5g,h), the NFM1Sb sample exhibits a significantly reduced intensity of the M-F (M = Ni/Fe/Mn) characteristic peaks in the F1s spectrum at 685–688 eV compared to the NFM, demonstrating that Sb doping effectively suppresses the dissolution of transition metals and their detrimental side reactions with fluorinated species in the electrolyte. Further tests on the metal dissolution of Ni, Fe, and Mn in the cycled pristine NFM and NFM1Sb samples were conducted, as shown in Figure 5i. Compared to NFM, the modified sample exhibits significantly suppressed dissolution of Ni, Fe, and Mn. These results collectively indicate that homogeneous Sb doping effectively inhibits detrimental interfacial side reactions and structural degradation, contributing to the enhanced electrochemical performance.

## 4. Conclusions

In summary, we synthesized Sb^3+^-doped, nickel–iron–manganese layered oxide cathode materials for sodium-ion batteries via a high-temperature solid-state reaction. XRD, TEM, XPS, and EDS analyses confirmed successful Sb doping, which optimized grain morphology, homogenized stress distribution, enhanced structural stability, and shortened Na^+^ diffusion pathways. Electrochemical tests revealed that Sb doping obviously reduced Na^+^ migration energy barriers and improved electronic conductivity. The optimized NFM1Sb (1%mol Sb) cathode achieved 86.48% capacity retention after 200 cycles at 1C and delivered 122.2 mAh g^−1^ at 5 C, while maintaining stable performance under high-voltage conditions. The facile synthesis and exceptional performance highlight its industrial potential, demonstrating that Sb doping-mediated grain morphology regulation effectively mitigates stress concentration, stabilizes interfaces, and enables long-cycle durability.

## Data Availability

The data that support the findings of this study are available upon request from the corresponding authors.

## Supplementary Materials

The following supporting information can be downloaded at https://www.mdpi.com/article/10.3390/nano15201575/s1, Figure S1: The SEM result of the four samples; Figure S2: The XRD result of the four samples; Figure S3: The refined XRD patterns of NFM0.5Sb and NFM2Sb; Figure S4: Line scan EDS of the cross-section of NFM1Sb sample; Figure S5: HRTEM image of NFM; Figure S6: Constant current charge–discharge curves of (a) NFM and (b) NFM1Sb at various cycle numbers; Figure S7: (a) Schematic diagram of the working principle of sodium-ion batteries. (b) Charge–discharge curves of NFM1Sb and hard carbon in half-cells. (c) Cycling performance of the full cell at 1 C; Figure S8: Impedance spectra of NFM and NFM1Sb after (a) 1 cycle and (b) 200 cycles at 1 C; Table S1. The crystal structure parameters of all samples were obtained through Rietveld refinement.
