# Supplementary material for: Enhancing Structural and Interfacial Stability of NaNi1/3Mn1/3Fe1/3O2 Cathodes via Sb3+ Doping for Sodium Ion Batteries
Source: Nanomaterials (Basel). 2025 Oct 16;15(20):1575. doi: 10.3390/nano15201575 (PMC12566254; doi:10.3390/nano15201575)
Supplement: Supplementary file 1 [file nanomaterials-15-01575-s001.zip › nanomaterials-3903640-supplementary.pdf]

Enhancing Structural and Interfacial Stability of $NaNi_{1/3}Mn_{1/3}Fe_{1/3}O_2$ Cathodes via Sb^{3+} Doping for Sodium Ion Batteries

Yong Liu [1,2], You Shi [1], Mengjie Zhang [1], Dan Sun [1], Huanhuan Li[3], Haiyan Wang [1,*], Yougen Tang [1,*]

1 Hunan Provincial Key Laboratory of Chemical Power Sources, College of Chemistry and Chemical Engineering, Central South University, Changsha 410083, China

2 Sunwoda Mobility Energy Technology Co., Ltd., Shenzhen 518100, China

3 School of Chemistry and Chemical Engineering, Henan Normal University, Xinxiang 453007, China

* Correspondence: wanghy419@csu.edu.cn; ygtang@csu.edu.cn;

Experimental section

Materials characterization

The crystal structure of the synthesized cathode materials was examined using X-ray powder diffraction (XRD) with a Rigaku Ultima IV diffractometer. Rietveld refinement was performed using GSAS software. The morphology, microstructure, and elemental composition of the samples were characterized using field-emission scanning electron microscopy (SEM, JSM-IT200, JEOL), high-resolution transmission electron microscopy (HRTEM, JEM-2100F, JEOL), and an attached energy-dispersive X-ray spectroscopy (EDS) system. The concentrations of different elements in the samples were determined by inductively coupled plasma optical emission spectrometry (ICP-OES, 8300, PerkinElmer). X-ray photoelectron spectroscopy (XPS, K-Alpha+, Thermofisher) was used to ascertain the valence states of various elements, using the C 1s peak at 284.8 eV as a reference. The instrument with QMG 220M analyzer was used to collect differential electrochemical mass spectrometry (DEMS) spectra. The DEMS system operates by coupling an electrochemical cell directly to a mass spectrometer via a gas-permeable membrane. As the battery material is being charged or discharged, volatile species (e.g., O_2, CO_2) generated at the electrode/electrolyte interface evolve into the cell's headspace. These gases are continuously transported by an inert carrier gas (e.g., Ar) into the high-vacuum chamber of the mass spectrometer. The mass spectrometer then ionizes the gas molecules and separates them according to their mass-to-charge ratio (m/z), allowing for the real-time, quantitative detection and identification of specific gaseous products. The key strength of DEMS is its ability to directly correlate the evolution of a specific gas (measured by its ion current) with the electrochemical reaction occurring at a specific voltage or capacity (measured by the potentiostat). This provides an unambiguous insight into the parasitic reactions, such as irreversible oxygen release from the lattice and subsequent electrolyte oxidation, which are critical for understanding the degradation mechanisms in battery materials.

Electrochemical measurements

Before electrochemical performance testing, the active material, conductive agent (Ketjen black), and binder (polyvinylidene fluoride) were mixed in a mass ratio of 8.6:0.7:0.7. An appropriate amount of N-methyl-2-pyrrolidone was added to this mixture, which was then coated onto an aluminum foil to serve as the electrodes. The electrodes were dried in a vacuum oven at 120 °C for 6 hours. They were then punched into disks with a diameter of 14 mm and calendared, with a mass loading of approximately 2.93 mg cm^2. Sodium metal and glass fiber (Whatman) were used as the counter electrode and separator, respectively. The electrolyte consists of 1 M NaPF6 in a mixture of ethylene carbonate (EC), propylene carbonate (PC), and dimethyl carbonate (DMC), with additives including sodium fluoro(ethylsulfonyl)imide (NaFSI), fluoroethylene carbonate (FEC), and diethyl sulfite (DTD). The half-cell was assembled based on a CR2016 coin cell. For the full cell, the anode active material was hard carbon (HC), and the HC anode was composed of 92 wt% active material, 3 wt% Super P, 1.5 wt% sodium carboxymethyl cellulose (CMC), and 3.5 wt% styrene-butadiene rubber (SBR). The anodes were coated on one side of a copper foil and electrochemically pre-sodiated using Na metal, and then paired with NFM1Sb cathode under a 2032-coin cell configuration with an N/P ratio of 1.05. Various charge/discharge performances and Galvanostatic Intermittent Titration Technique (GITT) tests were carried out using a LAND battery tester (CT2001A). The cyclic voltammetry (CV) characteristics of the electrodes were analyzed using a CHI760E electrochemical workstation. The CV curves at different scan rates were evaluated. Electrochemical Impedance Spectroscopy (EIS) measurements were conducted with an AC voltage amplitude of 10 mV, over a frequency range from 100 kHz to 10 mHz.

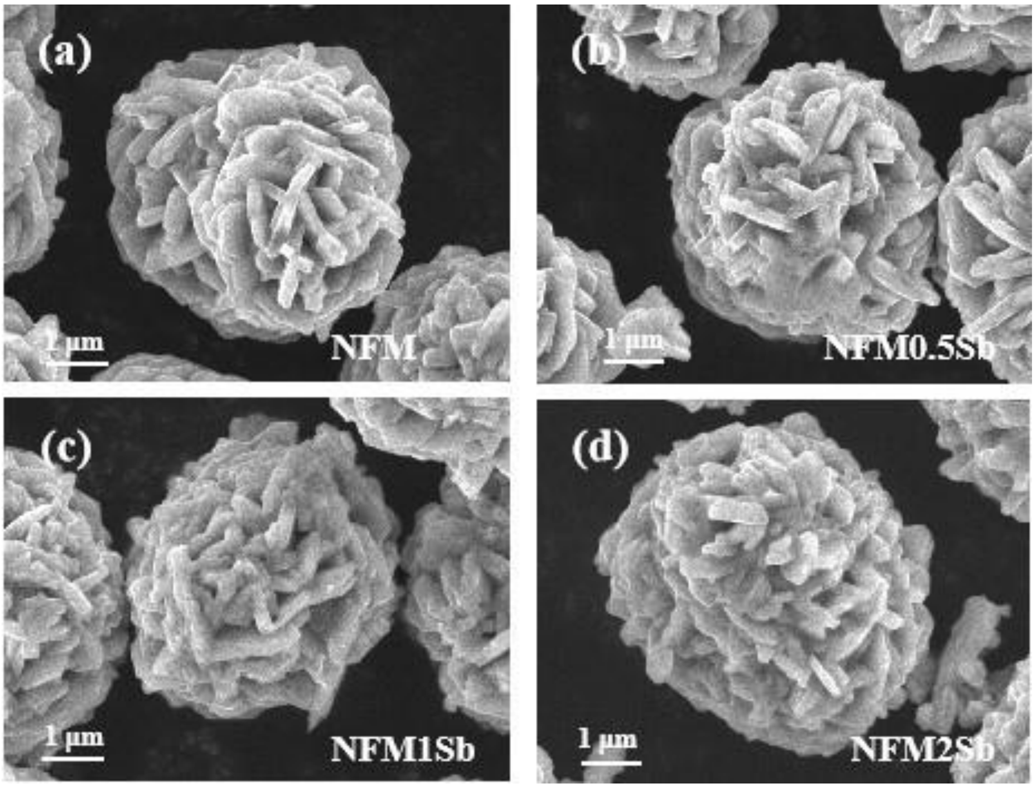

Figure S1. Microscopic morphology images of the four samples.

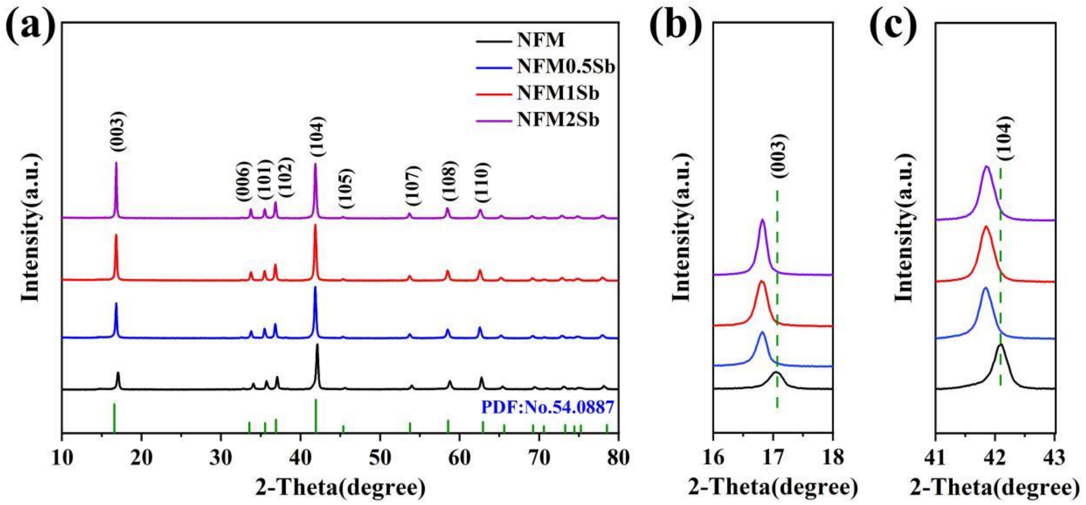

Figure S2. Powder XRD patterns of (a) NFM, NFM0.5Sb, NFM1Sb and NFM2Sb samples, magnified images of (b) the (003) and (c) (104) peaks.

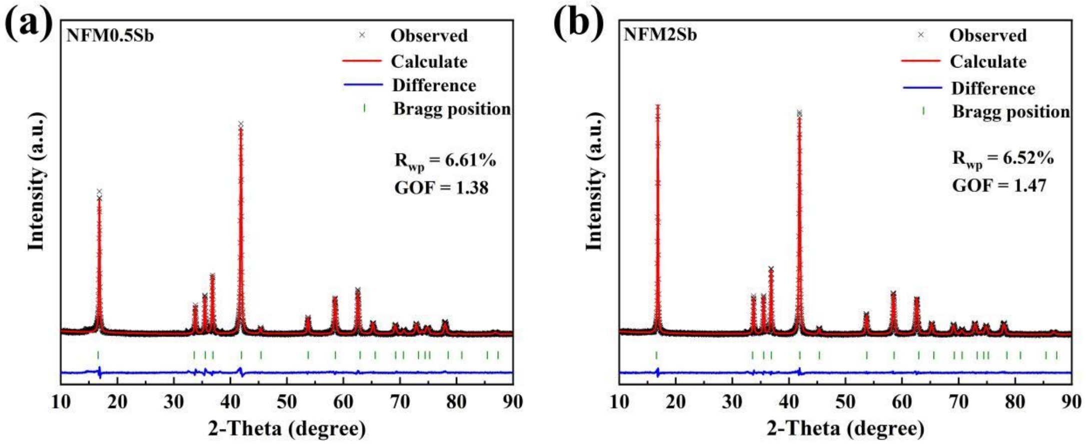

Figure S3. The refined XRD patterns of (a) NFM0.5Sb samples and (b) NFM2Sb samples.

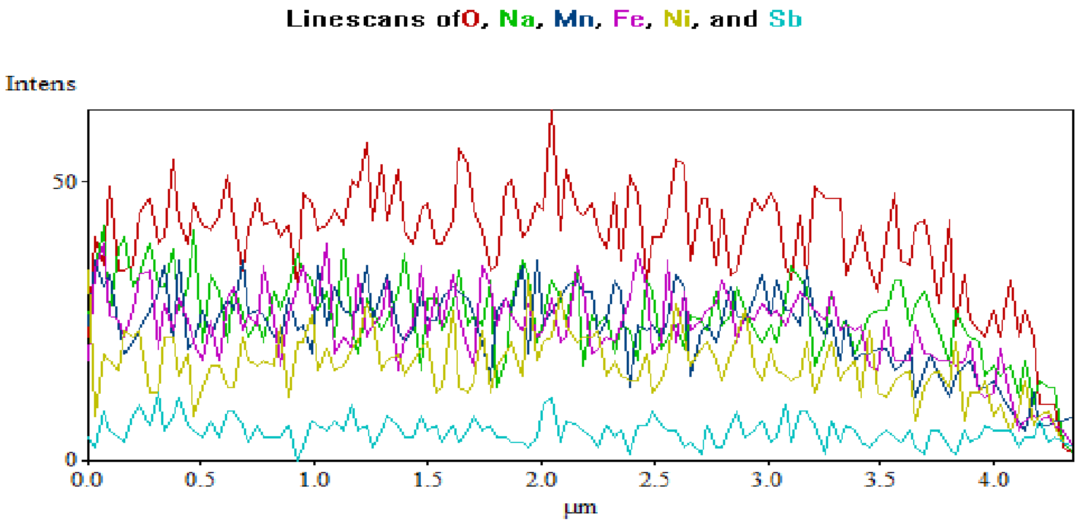

Figure S4. Line scan EDS of the cross-section of NFM1Sb sample.

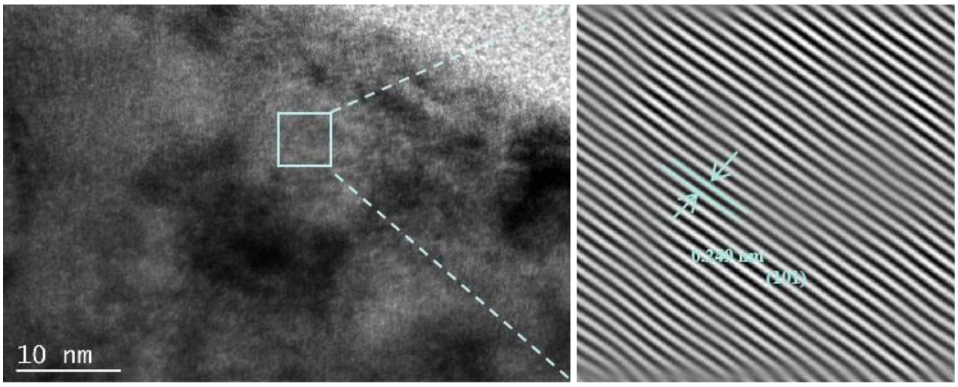

Figure S5. HRTEM image of NFM.

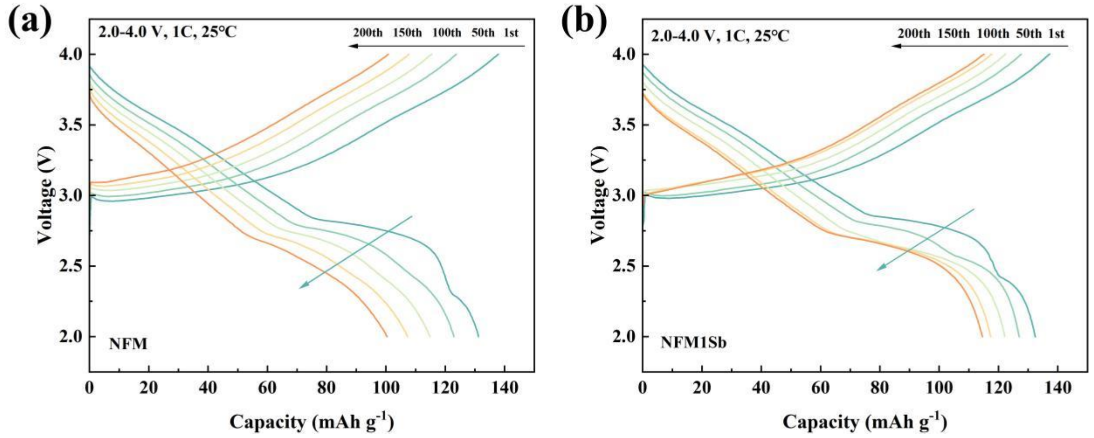

Figure S6. Constant current charge–discharge curves of (a) NFM and (b) NFM1Sb at various cycle numbers.

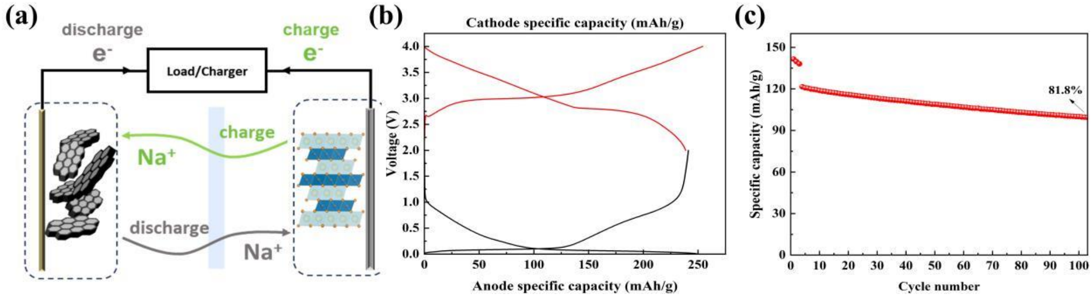

Figure S7. (a) Schematic diagram of the working principle of sodium-ion batteries. (b) Charge–discharge curves of NFM1Sb and hard carbon in half-cells. (c) Cycling performance of the full cell at 1 C.

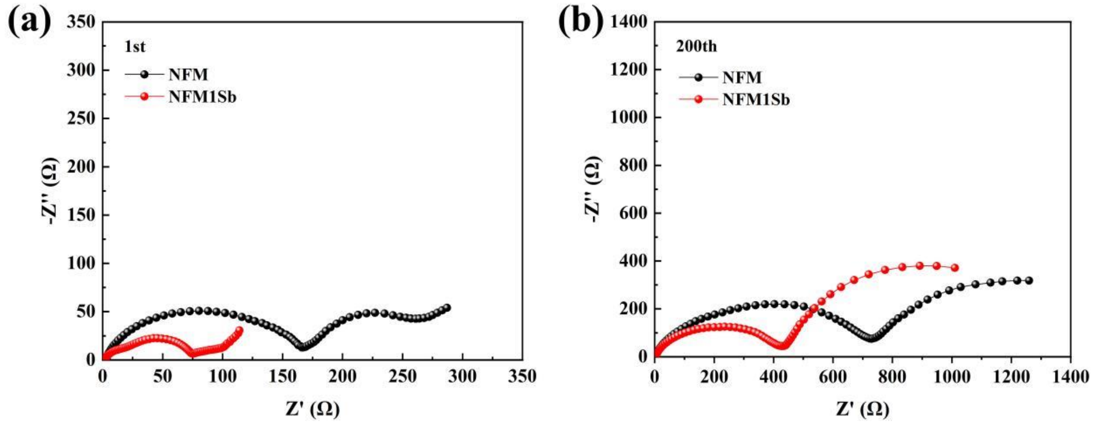

Figure S8. Impedance spectra of NFM and NFM1Sb after (a) 1 cycle and (b) 200 cycles at 1 C.

Table S1 The crystal structure parameters of all samples obtained through Rietveld refinement.

	a-axis(Å)	c- axis(Å)	V($Å^3$)	NaO_2(Å)	TMO_2(Å)	wR	GOF
NFM	2.97718	16.0053	122.858	2.99405	2.34104	6.574	1.46
NFM0.5Sb	2.98018	16.0124	123.161	2.99539	2.34209	6.613	1.38
NFM1Sb	2.98027	16.0219	123.241	2.99712	2.34345	6.193	1.37
NFM2Sb	2.98078	16.0348	123.381	2.99956	2.34533	6.523	1.47
